# Global Trends and Performances of Magnetic Resonance Imaging Studies on Acupuncture: A Bibliometric Analysis

**DOI:** 10.3389/fnins.2020.620555

**Published:** 2021-01-20

**Authors:** Jinhuan Zhang, Yangxin Zhang, Liyu Hu, Xingxian Huang, Yongfeng Liu, Jiaying Li, Qingmao Hu, Jinping Xu, Haibo Yu

**Affiliations:** ^1^The Fourth Clinical Medical College of Guangzhou University of Chinese Medicine, Shenzhen, China; ^2^Institute of Biomedical and Health Engineering, Shenzhen Institutes of Advanced Technology, Chinese Academy of Sciences, Shenzhen, China; ^3^Shenzhen Traditional Chinese Medicine Hospital, Shenzhen, China; ^4^CAS Key Laboratory of Human-Machine Intelligence-Synergy Systems, Shenzhen Institutes of Advanced Technology, Chinese Academy of Sciences, Shenzhen, China; ^5^School of Artificial Intelligence, University of Chinese Academy of Sciences, Beijing, China

**Keywords:** bibliometric analysis, Web of Science, co-citation analysis, acupuncture, magnetic resonance imaging

## Abstract

**Objectives:** To summarize development processes and research hotspots of MRI research on acupuncture and to provide new insights for researchers in future studies.

**Methods:** Publications regarding MRI on acupuncture from inception to 2020 were downloaded from the Web of Science Core Collection. VOSviewer 1.6.15 and CiteSpace V software were used for bibliometric analyses. The main analyses include collaboration analyses between countries/institutions/authors, co-occurrence analysis between keywords, as well as analyses on keyword bursts, citation references, and clusters of references.

**Results:** A total of 829 papers were obtained with a continually increased trend over time. The most productive country and institution in this field were the People's Republic of China (475) and KyungHee University (70), respectively. Evidence-based Complementary and Alternative Medicine (83) was the most productive journal, and Neuroimage (454) was the most co-cited journal. Dhond's et al. (2008) article (co-citation counts: 58) and Napadow's et al. (2005) article (centrality: 0.21) were the most representative and symbolic references, with the highest co-citation number and centrality, respectively. Jie Tian had the highest number of publications (35) and Kathleen K S Hui was the most influential author (280 co-citations). The four hot topics in MRI on acupuncture were acupuncture, fMRI, pain, and stimulation. The three frontier topics were connectivity, modulation, and fMRI. Based on the clustering of co-cited documents, chronic low back pain, sham electro-acupuncture treatment, and clinical research were the main research directions.

**Conclusion:** This study provides an in-depth perspective for MRI research on acupuncture and provides researchers with valuable information to determine the current status, hot spots, and frontier trends of MRI research on acupuncture.

## Introduction

Acupuncture is an ancient Chinese treatment that has been systematically used over 2000 years (Liang and Wu, 2006), and is now rapidly gaining popularity as a Western alternative to be used as a complementary practice for its undeniable therapeutic effects (Liang and Wu, 2006) in treating various pain-related and neurological conditions (Chon and Lee, 2013; Hao and Mittelman, 2014). However, in spite of its high acceptance, the neural mechanisms underlying acupuncture have not been well-understood.

In recent decades, neuroimaging technologies have provided a new perspective to improve our understanding of acupuncture mechanisms. Magnetic resonance imaging (MRI), due to its minimal invasiveness, lack of radiation exposure, excellent spatial resolution, and relatively wide availability, is widely used to explore how acupuncture affects the brain as well as the brain networks (Biella et al., 2001; Liu et al., 2004; Sun et al., 2014; Scheffold et al., 2015). With the increase of research in this area, several reviews have summarized relevant literatures (Huang et al., 2012; He et al., 2015; Scheffold et al., 2015; Cai et al., 2018), almost all of them were about specific mechanisms of acupuncture using functional magnetic resonance imaging (fMRI). However, little attention was paid to estimate the general situation and research trends in the MRI field of acupuncture.

Bibliometric analyses, a series of analyses for evaluating and quantifying literature information, have been applied in many research fields to identify the core researchers, institutions, and countries, as well as the cooperative relationship between them. Co-occurrence analysis of keywords, co-citation analysis, and burst of keywords can reflect the global research trends and topic hotspots (Chen et al., 2016; Leefmann et al., 2016; Ma and Ho, 2016). Lee et al. and Ma et al. performed a global bibliometric analysis based on the Web of Science database and PubMed to evaluate the development trends of acupuncture (Ma et al., 2016; Lee and Chae, 2019), respectively. However, a specific bibliometric analysis of MRI on acupuncture has not yet been performed. CiteSpace and VOSviewer software are characterized by co-occurrence network maps of authors, keywords, institutions, countries, and subject categories and co-citation networks of cited authors, cited references, and cited journals (Chen, 2006; van Eck and Waltman, 2010; Liu et al., 2015), which have been adopted to several studies (Liu et al., 2019; Yang et al., 2019; Chen et al., 2020; Qin et al., 2020).

Thus, in this study, CiteSpace and VOSviewer were applied to analyze the research situation, hot topics, and trends concerning MRI research on acupuncture over time with knowledge maps.

## Methods

### Data Acquisition

We did separate research from Web of Science and PubMed, resulting in 829 papers for Web of Science and 732 papers for PubMed from the date of their inception to October 2, 2020. Thus, we chose the results from the Web of Science Core Collection (WoSCC), since it contains more comprehensive literature.

We searched the Web of Science directly using the following two topics together: (1) (MRI OR magnetic resonance imaging OR resting state OR fMRI OR rs-fMRI OR functional connectivity OR task fMRI OR BOLD OR ReHo OR ALFF OR fALFF OR white matter OR voxel based analysis OR VBM OR voxel based morphometry OR Freesurfer OR surface based morphometry OR cortical thickness OR surface area OR cortical volume OR gray matter volume OR gray matter density), and (2) (acupuncture therapy OR acupuncture OR acupuncture point OR Acupuncture, Ear OR body acupuncture OR Auricular Acupuncture OR Electroacupuncture OR electroacupuncture OR Moxibustion) Indexes =SCI-EXPANDED, CCR-EXPANDED, IC Timespan = 1985-2020. As a result, 829 records were obtained.

### Analytical Tools

CiteSpaceV and VOSviewer 1.6.15, Java-based applications, were used to perform bibliometric analyses. VOSviewer (Netherlands' Leiden University) was used to identify journals, collaboration of countries and institutions, and co-authorship, keyword co-occurrence (van Eck and Waltman, 2010; Gao et al., 2018). The different nodes represent different countries, institutions, and keywords, while the size of the circle or font reflects the productivity. The link strength between nodes represented increased collaboration between countries, institutions, author and co-occurrence.

CiteSpace was used to identify centrality between countries/institutions, keyword bursts, citation reference bursts and clusters in this study (Chen, 2006, 2008). High centrality is often considered as a turning point or pivotal point in a field. The parameters of CiteSpace were set as follows: time slicing (1994–2020), years per slice (1), term source (all selection), node type (choose one at a time), and pruning (pathfinder). Detail information can be found at http://cluster.cis.drexel.edu/~cchen/citespace/ and https://www.vosviewer.com/.

## Results

### General Information for MRI Research on Acupuncture

#### Annual Publications and Document Type

**Figure 1:**
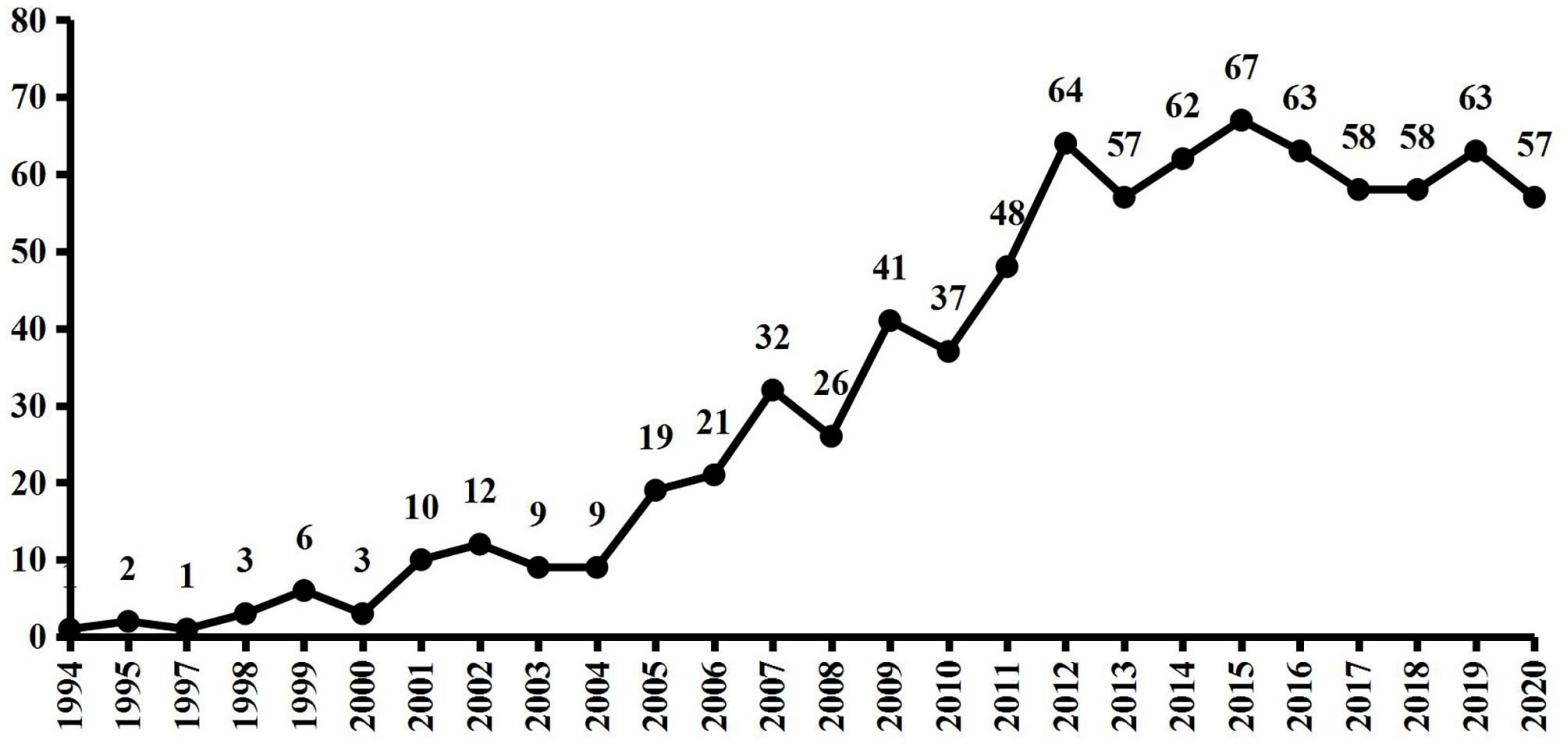
The number of MRI research works on acupuncture for publications indexed by SCI-E from 1994 to 2020. MRI, magnetic resonance imaging; SCI-E, Science Citation Index-Expanded.

The number of published works of literature each year is shown in Figure 1. As can be seen from the figure, the first paper was published in 1994. Although the number of studies fluctuated slightly from 1994 to 2012, the overall number was increasing gradually and reached the peak in 2012. In particular, the number of published works of literature fluctuated continuously from 2012 to 2020, but all of them were more than 50.

Ten document types were identified in a total of 829 references. The article, as the most popular document type, comprises 85.5% of the total production and was followed by reviews, editorial material, meeting abstracts, proceedings papers, corrections, letters, book chapters, retracted publications, and early access works (Table 1).

**Table 1:** Document types for documents on MRI research on acupuncture.

**Ranking**	**Type**	**Counts**	**(%)**
1	Article	709	85.5
2	Review	71	8.6
3	Editorial material	12	1.5
4	Meeting abstract	11	1.3
5	Proceedings paper	11	1.3
6	Correction	5	0.6
7	Letter	5	0.6
8	Book chapter	2	0.2
9	Retracted publication	2	0.2
10	Early access	1	0.1

#### Analysis of Country

**Figure 2:**
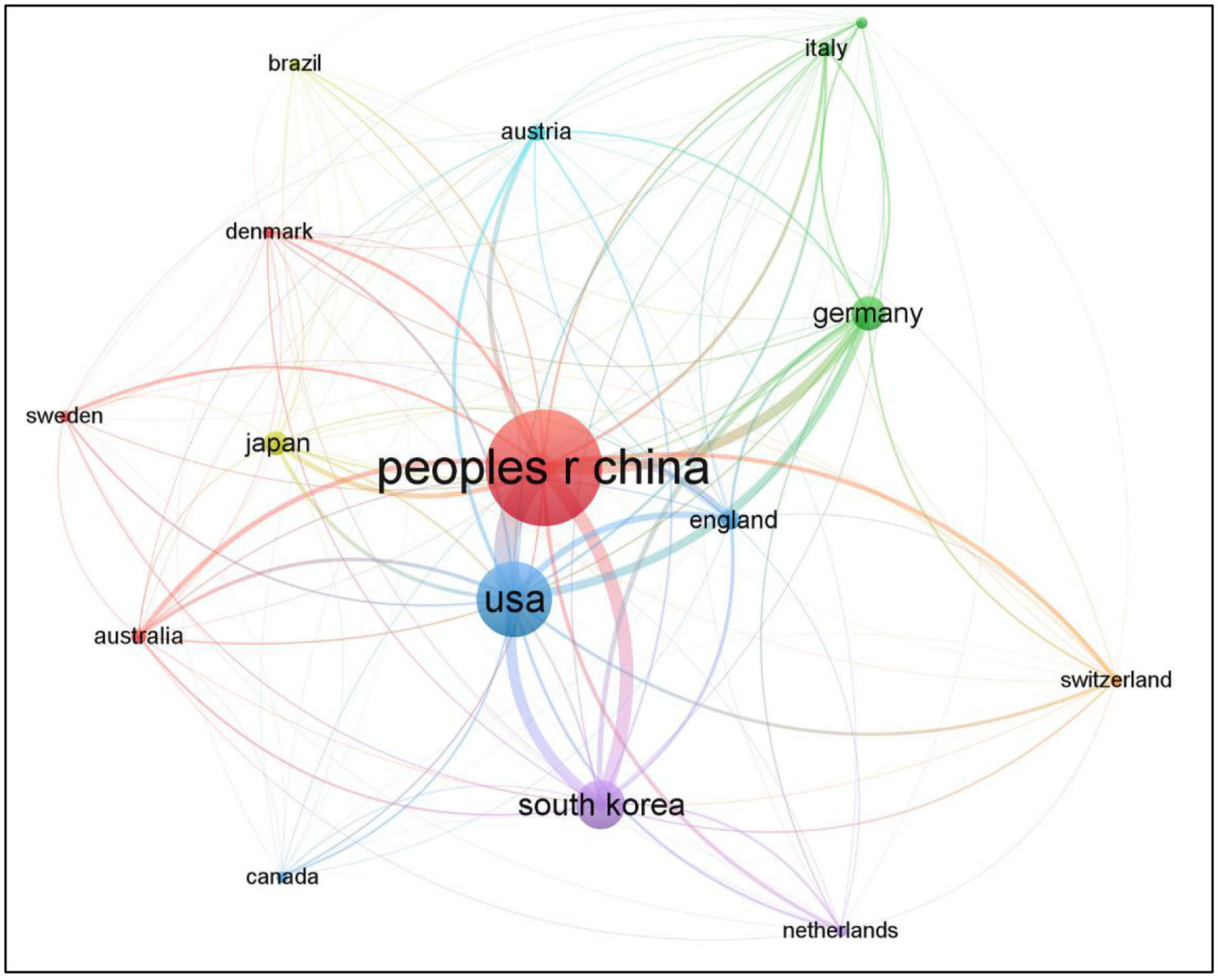
Map of active countries in MRI research on acupuncture. USA, United States of America.

Overall, the 829 references were published by 21 countries. For better visualization, we only selected 16 countries with more than 5 articles using VOSviewer (Figure 2). Each node represents a country, and the size of the node is proportional to the number of published articles. Connections between nodes represent collaborations, and the wider the connection, the tighter the collaboration.

The list of the top 5 countries were presented in Table 2. The People's Republic of China had the most publications, followed by the United States of America (USA), South Korea, Germany, and Japan. The top five countries in terms of centrality were USA (0.56), which had the highest centrality, followed by the People's Republic of China, Germany, South Korea, and Japan.

**Table 2:** Top 5 countries and institutions which performed MRI research on acupuncture.

**Ranking**	**Country/region**	**Frequency**	**Centrality**	**Institution**	**Abbreviations**	**Frequency**	**Centrality**
1	Peoples R China	475 (57.4%)	0.37	KyungHee University	KyungHee Univ	70	0.08
2	USA	228 (27.5%)	0.56	Chinese Academy of Sciences	Chinese Acad of Sci	64	0.08
3	South Korea	110 (13.3%)	0.08	Xidian University	Xidian Univ	56	0.03
4	Germany	60 (7.2%)	0.25	Massachusetts General Hospital	Massachusetts Gen Hosp	51	0.21
5	Japan	39 (4.7%)	0.04	Harvard University	Harvard Univ	44	0.14

*USA, United States of America*.

#### Analysis of Institution

**Figure 3:**
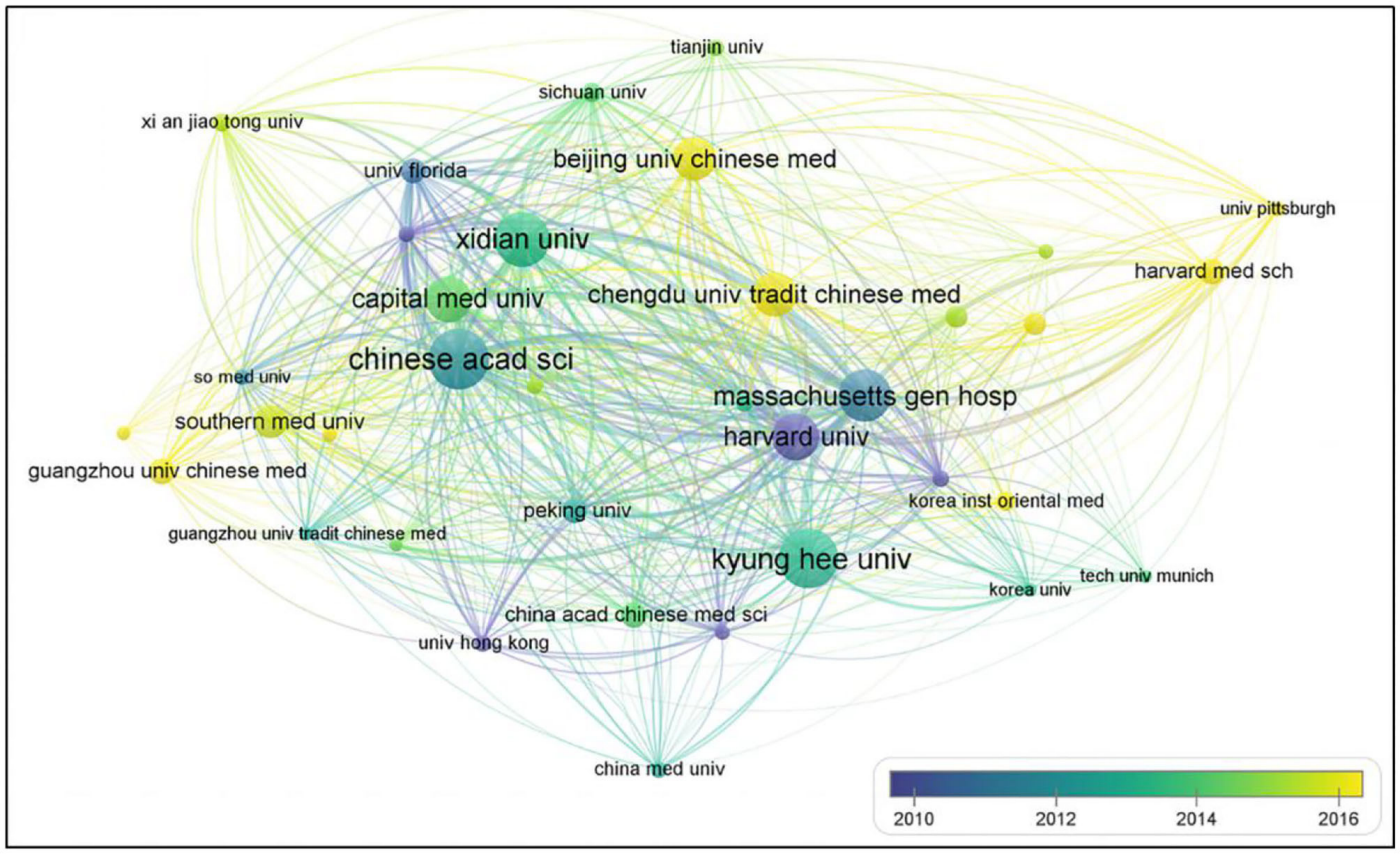
Map of active institutions on MRI research on acupuncture.

Nearly 197 institutions made contributions to MRI research on acupuncture. To obtain a better visualization, institution collaboration networks depict only 16 institutions, which had at least 20 papers using VOSviewer (Figure 3). Each node represents an institution, and the size of the node is proportional to the number of published articles. Connections between nodes represent collaborations, and the wider the connection, the tighter the collaboration.

The list of the top 5 institutions was presented in Table 2. KyungHee University has published the largest number of studies, followed by the Chinese Academy of Sciences, Xidian University, Massachusetts General Hospital, and Harvard University. Massachusetts General Hospital showed the highest centrality.

From the timeline of articles published by the institution using VOSviewer, Traditional Chinese Medicine (TCM) colleges, especially Chengdu University of TCM, Guangzhou University of Chinese Medicine(CM), and Beijing University of CM pay more attention to MRI research on acupuncture around 2016.

#### Analysis of Authors

**Figure 4:**
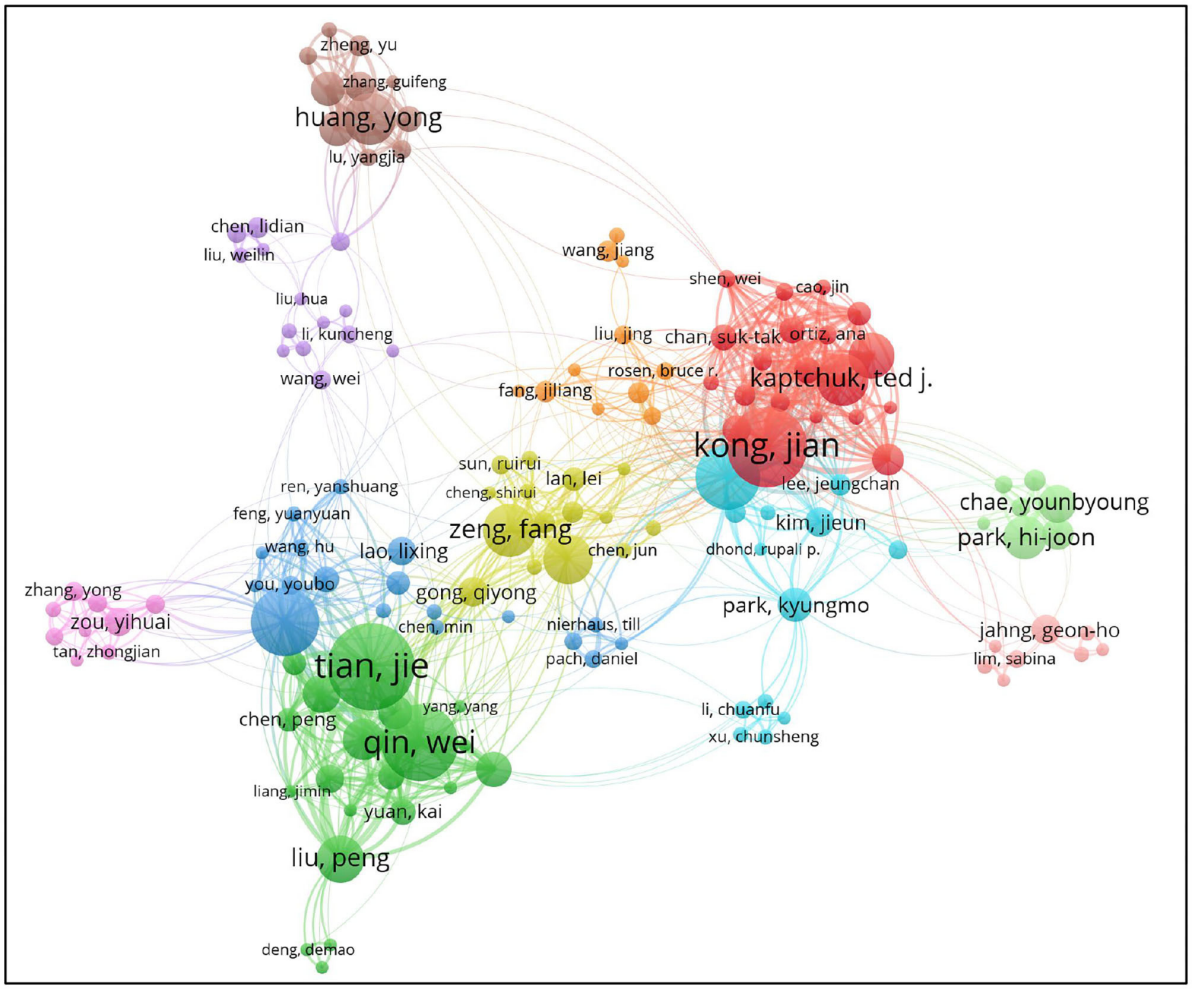
Map of authors' collaborations related to MRI research on acupuncture.

A co-author map was generated using VOSviewer, 829 publications were published by 429 research authors (Figure 4). Each node represents an author and the size of the node is proportional to the number of published articles. Connections between nodes represent collaborations and the wider the connection, the tighter the collaboration. Table 3 showed the top 10 authors who have published articles related to MRI research on acupuncture. They are active professional authors in the fields from China and USA, and their partnerships can be seen in an analysis of authors' collaborative networks using VOSviewer. Eleven clusters were formed, and each cluster contains authors who have long-term relationships.

**Table 3:** Top-10 authors who performed MRI research on acupuncture.

**Rank**	**Author**	**Frequency**	**Year**	**Country**
1	Tian Jie	44	2009	China
2	Kong Jian	40	2007	USA
3	Qin Wei	38	2009	China
4	Bai Lijun	33	2009	China
5	Napadow Vitaly	31	2007	USA
6	Zeng Fang	25	2013	China
7	Kaptchuk ted j	24	2007	USA
8	Huang Yong	24	2011	China
9	Liang Fanrong	23	2012	China
10	Liu Peng	22	2009	China

Among the top 10 authors, Kong Jian, Napadow Vitaly, and Kaptchuk ted j were from Harvard Medical School, Tian Jie and Qin Wei came from Chinese Academy of Sciences and Xidian University of China, Bai Liyun and Liu Peng came from Xi'an Jiaotong University. Zeng Fang and Liang Fanrong came from Chengdu University of TCM, Huang Yong came from Southern Medical University.

As can be seen from the publication year of the first article, the authors of Massachusetts General Hospital first published their MRI research on acupuncture, relevant studies by Chinese scholars were successively published since 2009.

#### Analysis of Journals and Co-cited Journals

**Figure 5:**
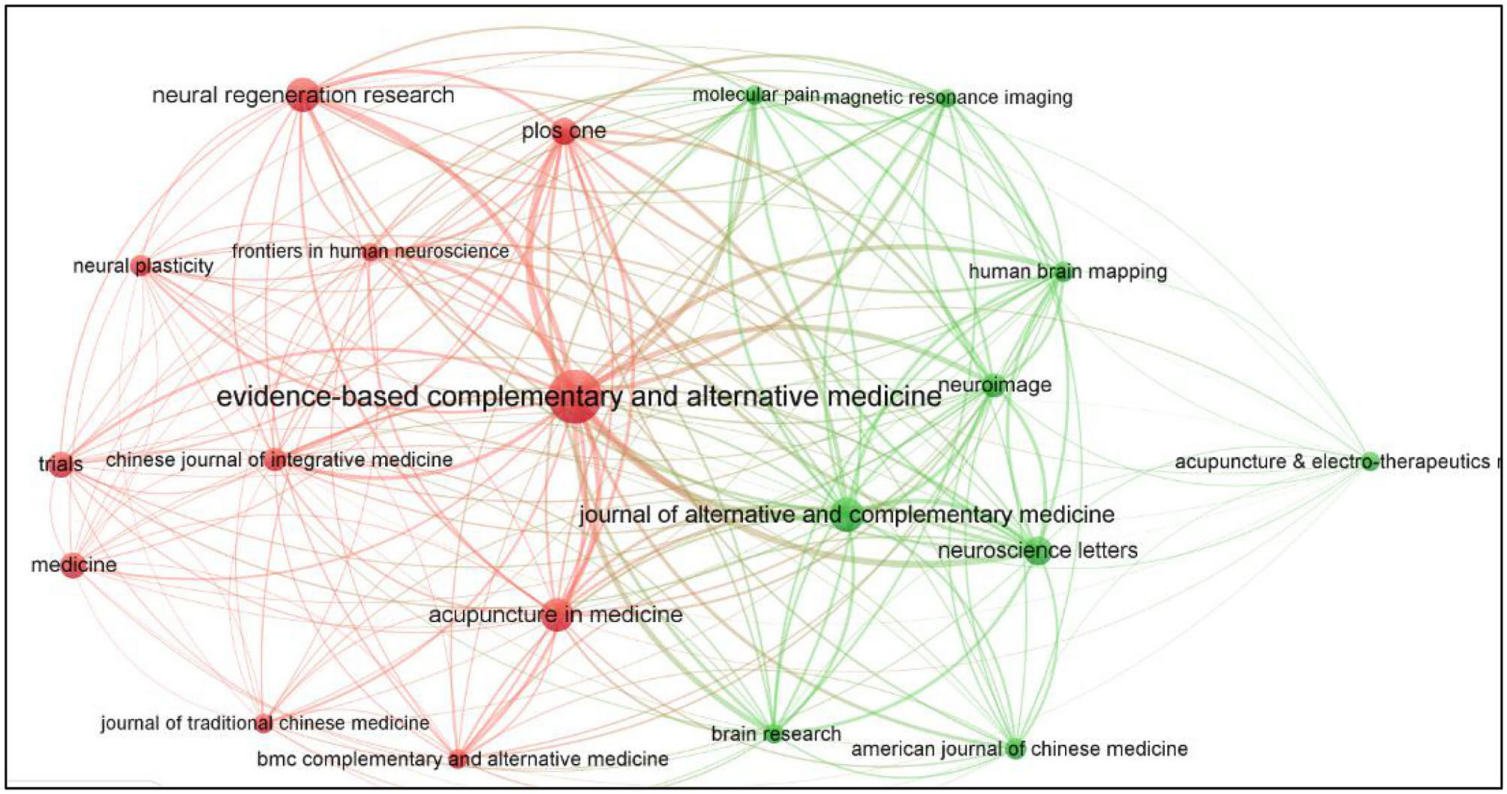
Map of journals on MRI research on acupuncture.

A total of 258 journals had published papers about using MRI on acupuncture, 20 journals of which had published over 10 papers (Figure 5).

Table 4 showed the top five scholarly journals that published articles related to MRI study on acupuncture. The first most productive journal was *Evidence-based Complementary and Alternative Medicine*, followed by *Journal of Alternative and Complementary Medicine, Neural Regeneration Research, Acupuncture in Medicine and Neuroscience Letters*. Among them, the average impact factor (IF) is 2.294.

**Table 4:** Top five journals and co-cited journals related to MRI research on acupuncture.

**Ranking**	**Journal**	**IF (Q)^**a**^2019**	**Frequency**	**Co-cited journals**	**Abbreviations**	**Centrality**	**Frequency**
1	Evidence-based Complementary and Alternative Medicine	1.813 (3)	83	NeuroImage	NeuroImage	0.08	454
2	Journal of Alternative and Complementary Medicine	2.109 (2)	35	Journal of Alternative and Complementary Medicine	J Altern and Complem Med	0	334
3	Neural Regeneration Research	3.171 (2)	34	Pain	Pain	0.14	322
4	Acupuncture in Medicine	2.129 (2)	33	Human Brain Mapping	Hum Brain Mapp	0.05	315
5	Neuroscience Letters	2.247 (3)	24	Neuroscience Letters	Neurosci Lett	0.09	291

a *IF and Q in category according to Journal Citation Reports (2019). IF, impact factor; Q, quartile*.

**Figure 6:**
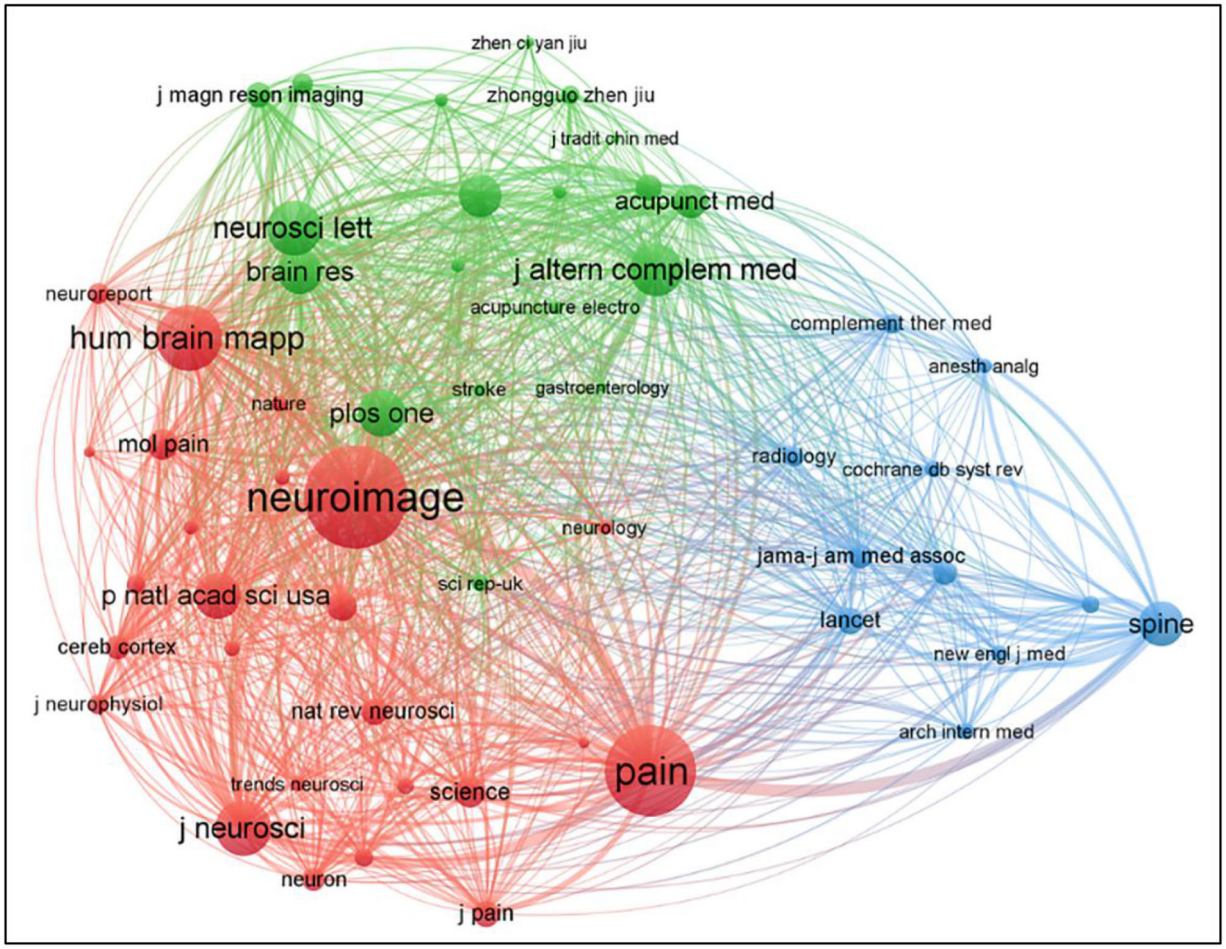
Map of cited journals on MRI research on acupuncture.

Figure 6 presented the 55 co-cited journals with at least 100 times. The top five co-cited journals were *Neuroimage, Journal of Alternative and Complementary Medicine, Pain, Human Brain Mapping*, and *Neuroscience Letters* (Table 5), among them, *Pain* has the highest centrality.

**Table 5:** Top five co-cited authors related to MRI research on acupuncture.

**Co-cited author**	**Abbreviations**	**Country**	**Frequency**	**Centrality**
Hui Kathleen K S	Hui KKS	USA	280	0.2
Kong Jian	Kong J	USA	188	0.05
Napadow Vitaly	Napadow V	USA	179	0.01
Bai Lijun	Bai LJ	China	122	0.02
Wu Mingting	Wu MT	China	109	0.03

### Research Hotspots

A research hotspot refers to a large number of interconnected papers or topics discussed in a certain period of time. Cited autors, co-occurring keywords, and cited references can be used to investigate current research hotspots.

#### Analysis of Co-cited Authors

**Figure 7:**
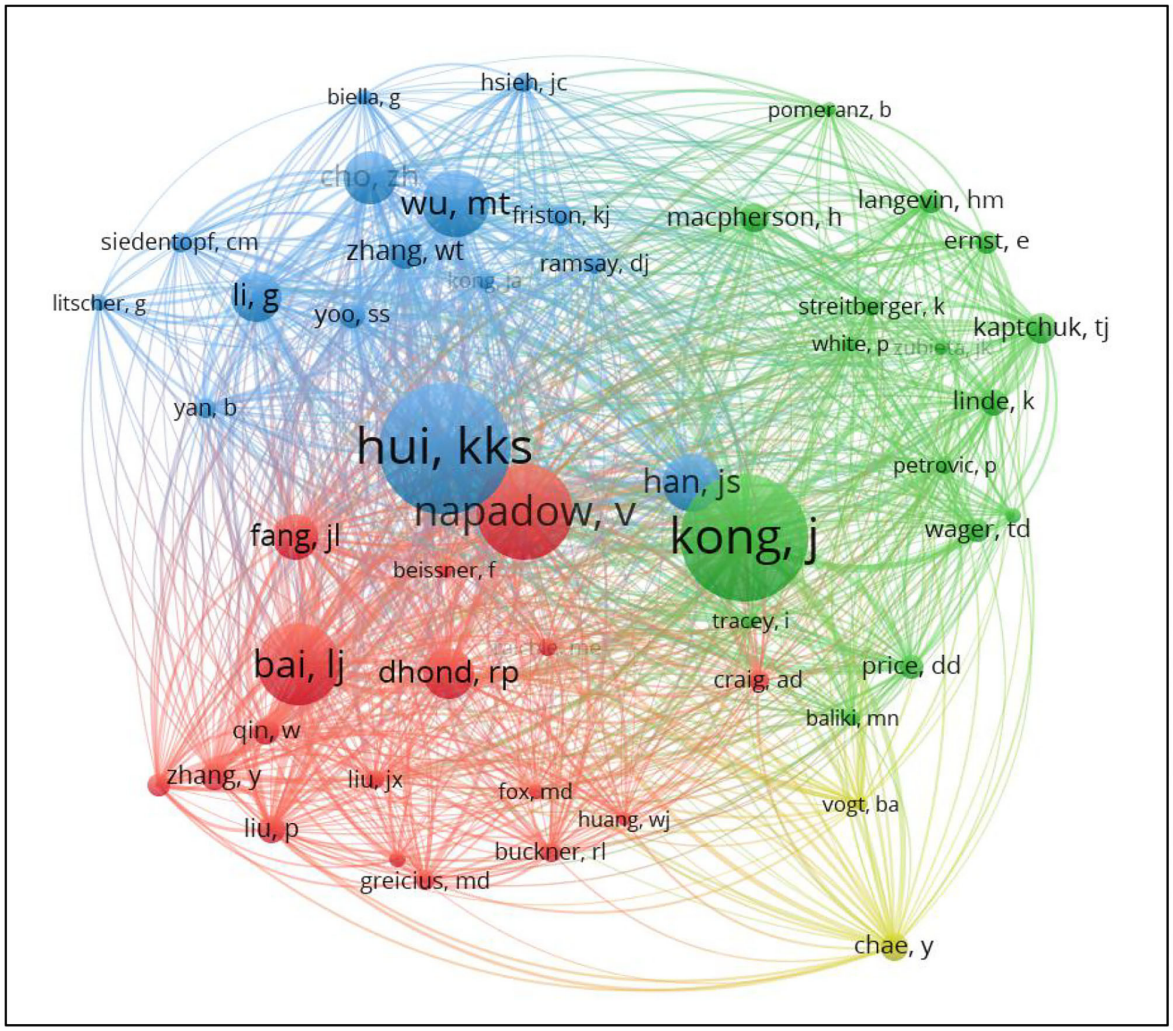
Map of co-cited authors related to MRI research on acupuncture.

The map of co-cited authors was presented in Figure 7. For better visualization, we only included 51 authors cited at least 50 times. Hui KKS ranked the first, followed by Kong Jian, Napadow V,Bai Lijun, and Wu Mingting. Among them, Hui KKS had the highest centrality (Table 5).

Hui KKS, Kong Jian, and Napadow V came from Massachusetts General Hospital. Among them, Hui KKS, and Napadow V were from the department of radiology, and Kong Jian was from the psychiatry department.

Bai Lijun, from Xi 'an Jiaotong University, is dedicated to computational neural imaging, image processing and pattern recognition, and information system modeling.

Wu Mingting was from institute of Traditional Medicine at National Yang-Ming University.

#### Analysis of Co-occurring Keywords

**Figure 8:**
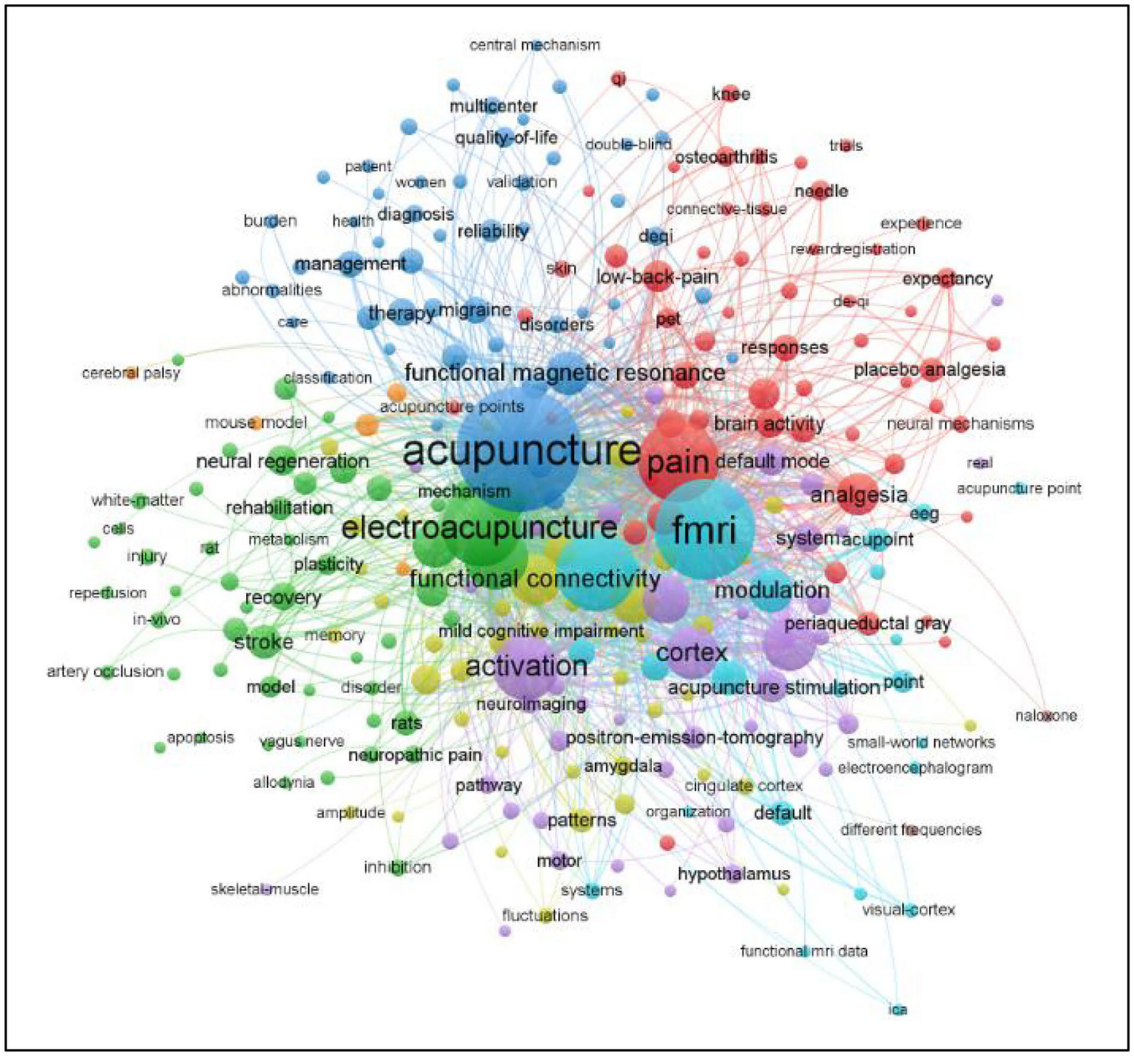
Map of co-occurring keywords related to MRI research on acupuncture.

An analysis in terms of co-occurrence frequency and centrality (Table 6; Figure 8) revealed that the maximum frequency was of “acupuncture” at 392, followed by “fMRI”(262), “pain”(161), “stimulation”(156), “electro-acupuncture”(155), “activation,” “brain,” “cortex,” “human brain,” and “connectivity.” Among them, “pain” has the highest centrality (0.14).

**Table 6:** Top 10 keywords related to MRI research on acupuncture.

**Ranking**	**Frequency**	**Keyword**	**Centrality**	**Ranking**	**Frequency**	**Keyword**	**Centrality**
1	392	Acupuncture	0.10	6	70	Activation	0.03
2	262	fMRI	0.03	7	61	Brain	0.02
3	161	Pain	0.14	8	24	Cortex	0
4	156	Stimulation	0.07	9	24	Human brain	0
5	155	Electroacupuncture	0.08	10	21	Connectivity	0

In terms of co-occurrence keyword clustering, it can be roughly divided into five clusters of different colors: Cluster 1 refers to burden of disease, with primary keywords of burden of disease, quality of life, and management. Cluster 2 refers to comforting effect of acupuncture with primary keywords of placebo analgesia and expectancy. Cluster 3 refers to diseases, with primary keywords stroke and mild cognitive impairment. Cluster 4 and 5 refer to mechanism of the brain to acupuncture using MRI, with primary keywords of modulation, activation, and functional connectivity.

#### Analysis of Co-cited References

A total of 21,598 references were generated from 829 records to an analysis of co-cited references. With a timespan from 1994 to 2020 and a time slice of one, the top five most cited or occurred items from each slice were chosen to form the network map of co-cited references using CiteSpace. Table 7 presents the top five co-cited references in relation to the co-citation counts. These five studies are all clinical studies, and the subjects are all healthy people.

**Table 7:** Top five cited references related to MRI research on acupuncture.

**Ranking**	**Co-citation counts**	**Co-cited reference**	**References**
1	57	Acupuncture modulates resting state connectivity in default and sensorimotor brain networks	Dhond et al., 2008
2	51	The integrated response of the human cerebro-cerebellar and limbic systems to acupuncture stimulation at ST 36 as evidenced by fMRI	Hui et al., 2005
3	49	The salient characteristics of the central effects of acupuncture needling: limbic-paralimbic-neocortical network modulation	Fang et al., 2009
4	49	Acupuncture modulates spontaneous activities in the anticorrelated resting brain networks	Bai et al., 2009
5	45	Effects of electroacupuncture versus manual acupuncture on the human brain as measured by fMRI	Napadow et al., 2005

The first article was published in 2008 by Dhond et al. (2008), which demonstrated that acupuncture can enhance the spatial extent of the post-stimulation resting brain network, including the regions of anti-nociceptive, emotion, and memory.

The second article was published in 2005 by Hui et al. (2005) with the highest centrality. This is the first study to show that acupuncture regulates neural activity at multiple levels of the cerebral-cerebellum and limbic system, which plays an important role in the neural mechanism of acupuncture.

The third article was published in 2009 by Fang et al. (2009), which used Taichong (LV3), Xingjian (LV2), Neiting (ST44), and a sham point on the dorsum of the left foot as targets, and showed that acupuncture can modulate the limbic-paralimbic-neocortical network.

The fourth article was published in 2009 by Bai et al. (2009), which used the non-repeated event-related fMRI (NRER-fMRI) design, to investigate such prolonged effects after acupuncture. This study showed that verum acupuncture enhanced dichotomy in the central-executive network (CEN) and DMN networks.

The last article was published in 2005 by Napadow et al. (2005), which compared the central effects of electro-acupuncture and manual acupuncture with fMRI. Results showed that the limbic system is the center of the acupuncture effect and has nothing to do with the specific acupuncture method.

### Global Trends in MRI Research on Acupuncture

#### Analysis of Keywords With the Strongest Citation Bursts

“Burst words” represent keywords that are cited frequently over a period of time, thereby indicating the frontier areas. As shown in Table 8, the green line represents the time period from 1994 to 2020, while the periods of each burst keyword are plotted by the red line. Keywords with citation bursts first appeared in 2009. The four highest strength burst keywords were “connectivity,” “modulation,” “fMRI,” and “default mode network.”

**Table 8:** Top 20 keywords with the strongest citation bursts.

**Keywords**	**Year**	**Strength**	**Begin**	**End**	**1994–2020**
Connectivity	1994	8.08	2012	2020	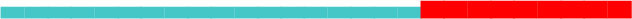
Modulation	1994	7.84	2011	2020	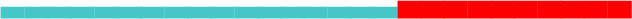
fMRI	1994	6.38	2011	2020	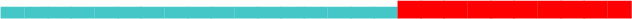
Default mode network	1994	6.12	2016	2020	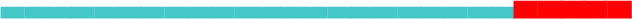
Nerve regeneration	1994	5.83	2014	2020	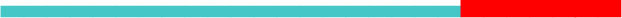
Manual acupuncture	1994	5.63	2012	2020	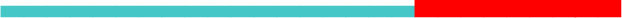
Efficacy	1994	5.42	2016	2020	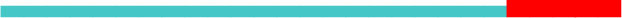
Default mode	1994	5.38	2009	2020	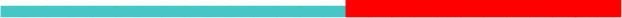
Network	1994	5.33	2013	2020	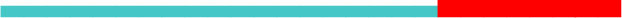
Meta analysis	1994	5.23	2015	2020	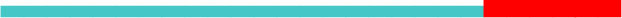
Neural regeneration	1994	4.94	2011	2020	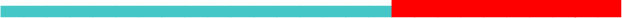
Brain network	1994	4.81	2012	2020	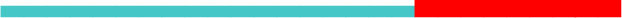
Default	1994	4.54	2010	2020	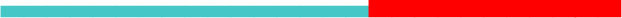
Recovery	1994	4.53	2015	2020	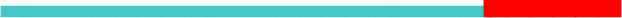
Disease	1994	4.4	2014	2020	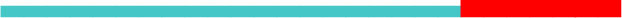
Prevalence	1994	4.31	2016	2020	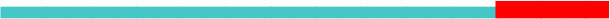
Resting state	1994	3.94	2010	2020	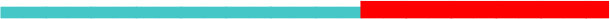
Functional mri	1994	3.86	2009	2020	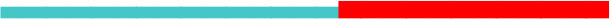
Therapy	1994	3.83	2015	2020	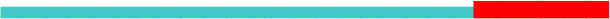

#### Analysis of Reference With the Strongest Citation Bursts

Significant increases of research interests in the MRI on acupuncture field are highlighted by publications with citation bursts. Table 9 showed the top 20 references with the strongest citation bursts during the period between 1994 and 2020. The red bars mean references cited frequently, and the green bars were references cited infrequently. Burst references refer to references heavily cited by articles over a period of time. From the bursts of cited references, the literature from 1999 to 2007 has opened the research hotspot of MRI on acupuncture.

**Table 9:** Top 20 references co-citation with the strongest citation bursts.

**References**	**Year**	**Strength**	**Begin**	**End**	**1994–2020**
Hui et al. (2005)	2005	24	2007	2020	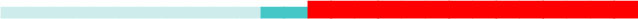
Napadow et al. (2005)	2005	18.79	2007	2020	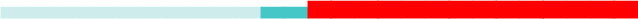
Hui et al. (2000)	2000	17.22	2001	2020	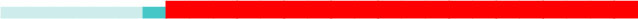
Wu et al. (2002)	2002	14	2003	2020	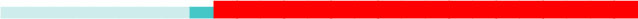
Wu et al. (1999)	1999	11.5	2001	2020	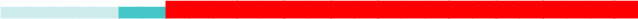
Cho et al. (1998)	1998	11.25	1999	2020	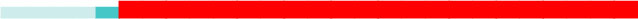
Yan et al. (2005)	2005	10.87	2007	2020	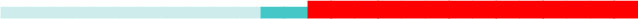
Yoo et al. (2004)	2004	9.21	2005	2020	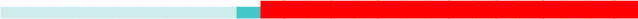
Biella et al. (2001)	2001	9.19	2002	2020	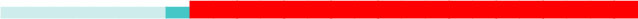
Hsieh et al. (2001)	2001	8.11	2002	2020	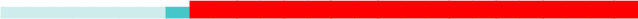
Liu et al. (2004)	2004	7.44	2007	2020	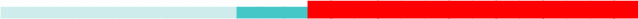
Zhang et al. (2003)	2003	7.33	2005	2020	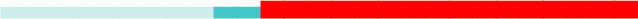
Siedentopf et al. (2002)	2002	7.18	2003	2020	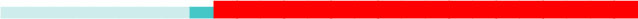
Kong et al. (2002)	2002	7.18	2003	2020	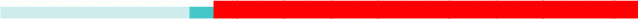
Parrish et al. (2005)	2005	7.17	2006	2020	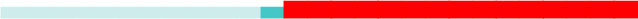
Napadow et al. (2007)	2007	7.16	2007	2020	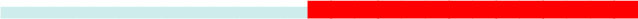
Dhond et al. (2007)	2007	7.08	2008	2020	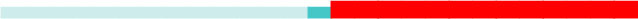
Kong et al. (2006)	2006	7.03	2007	2020	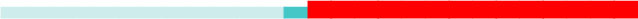
Kong et al. (2005)	2005	6.14	2007	2020	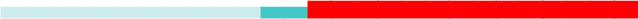
Wager et al. (2004)	2004	6.02	2006	2020	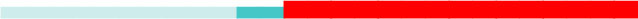

References with citation bursts first appeared in 1998. Most of the references had citation bursts between 2000 and 2007. The authors of the first four strongest burst references are, respectively, Hui KKS, Napadow V, and Wu MT. The strongest burst starting from 2005 was from Hui KKS and her team (Hui et al., 2005), which revealed that acupuncture induced a comprehensive response of the cerebral-cerebellar and limbic system when ST 36 was stimulated.

To get the key cluster of cited references, three specialized metrics—TFIDF, log-likelihood tests (LLR) and mutual information tests (MI) were used. With a modularity Q of 0.8726 and a means silhouette of 0.9864, the map consisted of 557 nodes and 1,627 links. The value of modularity Q and means silhouette suggested the clusters were rational. LLR usually gives the best result in terms of the uniqueness and coverage of themes associated with a cluster. The detailed information of the 7 clusters was summarized in Table 10.

**Table 10:** Top-ranked clusters on MRI research on acupuncture.

**Cluster ID**	**Size**	**Silhouette**	**Label (TFIDF)**	**Label (LLR)**	**Label (MI)**	**Mean (Cite Year)**
0	228	0.907	Acupuncture	Chronic low back pain	Central nociceptive coding	2013
2	156	0.892	Acupuncture	Sham electroacupuncture treatment	Various brain area	2007
3	141	0.978	Clinical research on acupuncture	Clinical research	Functional connectivity	2000
4	113	0.962	Chronic low back pain	Chronic low back pain	Structural properties	2015
5	77	0.976	Preliminary experience	Central nervous pathway	Functional connectivity	1997

The largest cluster was “chronic low back pain,” consisting of 228 references. The silhouette of this cluster was 0.907, which showed that the result was meaningful. The most active citers to this cluster was a review published by Qiu et al. (2016), who reviewed the PubMed Database during 1995–2014 on acupuncture-neuroimaging studies. This review suggests that future studies should focus on the quality control of acupuncture, so as to improve the reproducibility and reliability of neuroimaging studies of acupuncture.

The second cluster was “sham electroacupuncture treatment” with a silhouette of 0.978 and a member of 141. The most active citer to the cluster was a review published by Colloca et al. (2008), which systematically reviewed the literature that deals with placebo analgesia, emphasizing both the methodological aspects and the neurobiological advances.

The third cluster was “clinical research” with a silhouette of 0.892 and a member of 156. The most active citer to the cluster was a review published by Birch et al. (2004), which found that evidence of efficacy of acupuncture has apparently reached a sufficient threshold to draw a firm conclusion from well-designed studies, although all reviews agree that methodological rigor in clinical trials of acupuncture is generally poor.

## Discussion

Bibliometric analysis of MRI research on acupuncture using CiteSpace and VOSviewer from 1994 to 2020 was performed. We summarized the general information, research hotspots and research trends on MRI research on acupuncture.

### General Information for MRI Research on Acupuncture

As can be seen from the annual publication about MRI research on acupuncture, it has been kept in a relatively stable state and has not seen a great breakthrough in the last few years. This phenomenon may be related to the complexity of acupuncture effect, interdisciplinary or acupuncture only as complementary medicine and so on, but it also indicates that there is a great potential in the future.

The output countries are mainly from the USA and China. China published the most articles, suggesting that acupuncture, an ancient Chinese treatment, is widely accepted in China. The USA is a central collaborator with other countries, as indicated by its highest centrality. The output institutions are mainly from Massachusetts General Hospital and Harvard University of USA, Chinese Academy of Sciences, Xidian University of China, and Kyung Hee University of South Korea. The authors are mainly Jian Kong of Massachusetts General Hospital and Harvard University, Jie Tian and Wei Qin of Xidian University. Most of them are from MRI or psychological backgrounds. In addition, the USA and its two institutions occupy a central position and maintain a high degree of cooperation with countries and institutions. Collaboration helps researchers who investigated MRI research on acupuncture share resources and exchange knowledge and ideas, which is crucial for further development of the MRI research on acupuncture. Thus, stronger collaboration networks should be established among more countries, institutes, and authors, especially in China.

Although acupuncture originated in China, there are more than 20 TCM universities in China, and their publications is not as many as that of other institutions. It may be related to cross-disciplines, but also related to the fact that most acupuncturists in China pay more attention to clinical efficacy. Interestingly, Universities of TCM are becoming increasingly aware of this phenomenon and are gradually devoting themselves to MRI research on acupuncture. In the future, it may be more conducive to the development and spread of acupuncture, if researchers specializing in acupuncture therapy may focus more on the combination of relatively subjective clinical efficacy and objective indicators of brain imaging, more importantly, strengthen cooperation with experts from other professional backgrounds.

Articles about MRI research on acupuncture were published in 258 different journals, and the top 5 journals published 25% of publications. The top two journals are *Evidence-based Complementary and Alternative Medicine* and *Journal of Alternative and Complementary Medicine*. This indicated that acupuncture therapy is still regarded as complementary and alternative medicine, and as a result, relevant articles are mainly published in these two journals. Among the 57 articles published in 2020, only 3 articles (Cao et al., 2020; Kim et al., 2020; Tu et al., 2020) of IF(2019)>5, and the first author were all from Massachusetts General Hospital and Harvard Medical School, and the research topics were all about the effects of acupuncture on pain, which revealed MRI research on acupuncture for pain is a hot spot and has been accepted by high-impact journals.

In the future, as acupuncture is gradually recognized and accepted. The premise is that acupuncture does have significant advantages over other interventions in some diseases such as pain. Research with good design and quality control should be published in high-quality journals gradually, which is conducive to exchange of research findings on MRI research on acupuncture.

### Research Hotspots for MRI Research on Acupuncture

The current research hotspots of MRI research on acupuncture were investigated from the three aspects of co-cited authors, keywords, and cited references, which help researchers explore the distribution of topics within a particular academic discipline (Zhou et al., 2018).

In terms of co-cited authors, Hui KKS, Jian Kong, and Napadow V have long-term cooperation with each other (Napadow et al., 2005, 2007, 2009; Hui et al., 2010; Kong et al., 2018). Napadow V and Hui KKS often cooperated in acupuncture effects on the human brain by fMRI such as deqi, placebo analgesia, and default mode, especially acupuncture effects on the limbic-paralimbic-neocortical network (LPNN). Jian Kong mainly focused on the expectancy manipulation model, which refers to how expectation can significantly modulate pain perception due to his psychiatry background. Indeed, how to select the proper control group to reduce the placebo effect has been controversial in this field of research.

Our keyword co-occurrence analysis revealed that the three most commonly used keywords are acupuncture, fMRI, and pain. Pain is a painful disease process that can lead to a reduced quality of life and increased health care costs. The Institute of Medicine (IOM) reports that 100 million adults suffer from chronic pain, which costs about $600 billion a year (Dzau and Pizzo, 2014). Medication may only partially relieve the chronic pain and can be associated with unwanted side effects (Nahin et al., 2016). As a result, many people turn to complementary and alternative therapies such as acupuncture as part of their pain management. Several systematic reviews also have shown that Acupuncture has a good effect in alleviating pain and improving the quality of life (Manyanga et al., 2014; MacPherson et al., 2017; Smith et al., 2020). However, its therapeutic mechanisms remain controversial, partly because of the absence of an objective way of measuring subjective pain. The development of fMRI has brought light to the study of pain mechanism by acupuncture, which has been the focus of research in recent years. Moreover, several previous systematic reviews suggested that acupuncture can regulate the activity of multiple cortical and subcortical brain regions (Huang et al., 2012; Sun et al., 2014; Villarreal et al., 2016). The most common areas involved are the anterior cingulate cortex (ACC), amygdala, insular cortex, primary somatosensory cortex (S1), secondary somatosensory cortex (S2), thalamus, and prefrontal cortex (PFC), which overlap the pain matrix (Apkarian et al., 2005). A recent study (Kim et al., 2020) suggests that acupuncture may improve the tactile sensitivity of the pain affected area by regulating the somato-specific structure S1 neuroplasticity, which strengths the previous conclusion.

Our clustering analysis of keywords revealed five focus areas of MRI research on acupuncture using VOSviewer. It mainly involves the diseases currently studied, such as stroke, cognitive impairment and pain, etc., the burden on the society, the MRI mechanism of acupuncture on diseases and comforting effect of acupuncture. The above may indicate that the current main research is still on the MRI mechanism and comfort effect of acupuncture on some chronic diseases.

According to the five references cited most, acupuncture modulated multiple brain networks, including DMN, sensorimotor network (SMN), CEN, and LPNN. This is consistent with the results of a meta-analysis of fMRI studies after acupuncture stimulation (Chae et al., 2013). In addition, the mode of acupuncture may have no obvious correlation with brain functional response, while the sensation of *deqi* is correlated with brain function.

Therefore, combined with the findings of this study and current reviews about MRI research on acupuncture, acupuncture could not only activate the sensorimotor brain region and cause extensive inactivation of the limb-parietal neocortex network, but also modulate the connectivity of several brain regions, including DMN, SMN, CEN, and LPNN, which was associated with pain, emotion and memory (He et al., 2015). Verum acupuncture and sham acupuncture showed different brain responses. Compared with sham acupuncture, verum acupuncture significantly enhanced connectivity between ACC, left posterior cingulate cortex (PCC), insula, marginal/parietal margin, and precuneus (Cai et al., 2018). Deqi is considered as an important factor affecting the clinical efficacy of acupuncture. One study by Hui KKS revealed that acupuncture with deqi induced extensive deactivation and activation (Hui et al., 2010) in different brain regions. However, because of the difference between deqi and mixed sensations, subjects with severe pain should be excluded when exploring the neural mechanism of acupuncture stimulation (Sun et al., 2012). There was no significant difference in brain connection between acupuncture and electroacupuncture, especially for DMN (Jiang et al., 2013).

### Global Trend for MRI Research on Acupuncture

The global trend for MRI research on acupuncture was investigated from the strongest citation bursts of keywords and references as well as the clusters of references.

Keyword bursts may indicate frontier topics or emerging trends (Zhou et al., 2018). Connectivity, fMRI, modulation, and DMN are the four strongest citation bursts of keywords. In recent years, researchers have focused more on relationships between brain regions on the mechanism of acupuncture instead of a single brain area (Cai et al., 2018). The role of brain connectivity is going to be crucial and influential in the fields of neuroscience and medicine over the coming years (Edison, 2020). Brain connectivity analysis aims to characterize information propagation relationship in multichannel neural signals, which indicates underlying structural and functional relationship between regions of brain (Friston, 2011). One review (Cai et al., 2018) included 44 neuroimaging studies about acupuncture on functional connections in the brain, which showed that acupuncture increased connectivity of the default mode network (DMN) and sensorimotor network (SMN) in areas of the brain associated with pain, emotion and memory. Verum acupuncture significantly enhanced connectivity in some brain areas, compared with sham acupuncture.

Accumulating fMRI evidence has demonstrated that acupuncture is effective for a variety of diseases because it can modulate brain functional networks and brain activity (Fang et al., 2009; Feng et al., 2011; Wang et al., 2014). A review (Xiao et al., 2018) of the plasticity modulation of the nervous system by acupuncture suggested that modulation of neuroplasticity by acupuncture may be related to the effects of acupuncture on neurotrophins and neurotransmitters.

fMRI is an imaging method developed to show time-varying changes in brain metabolism (Ogawa and Lee, 1990; Bandettini et al., 1992; Kwong et al., 1992). fMRI, a non-invasive imaging technique, has been used to observe the human brain response to acupuncture stimulation since the mid-1990s (Cho et al., 1998). This technology provides us with information more directly involved in the anatomical and physiological function of acupuncture (Liu et al., 2009), revealing that the mechanism of acupuncture is mediated by the central nervous system (Qin et al., 2008; Kang et al., 2013).

Default mode network (DMN) is a functional network that is active at rest and immune to external stimuli (Ingvar, 1979; Raichle et al., 2001). One study found that brain areas within DMN overlapped largely with acupuncture response areas (Chae et al., 2013), which led the researchers to hypothesize that acupuncture works by regulating DMN (Otti and Noll-Hussong, 2012; Zhao et al., 2014). Clinical studies have observed that acupuncture modulated DMN connectivity in some diseases, such as pain (Li et al., 2014), depression (Quah-Smith et al., 2013), and Alzheimer disease (Liang et al., 2014).

Authors of the first three highest strength burst reference were all from Massachusetts General Hospital and Harvard Medical School in the past decade. Strikingly, all three papers support the conclusion that the limbic system is central to the effect of acupuncture. The cluster analysis of references first divides them into three main categories: clinical research, sham electroacupuncture treatment, and chronic low back pain. The implications of the clustering analysis of references are that the design and quality control of clinical studies are very important, as can be seen by analyzing the most active citer.

Although acupuncture has an important modulation on functional connections of the brain, especially DMN, as noted in a review of the modulation of DMN by acupuncture, whether this modulation is the core mechanism of acupuncture treatment remains unclear (Zhang et al., 2019). Just as one activation likelihood estimation (ALE) meta-analysis (Chae et al., 2013) of fMRI studies on acupuncture found that the brain's hemodynamic response to acupuncture reflects not only sensory discrimination but also the cognitive and emotional aspects of pain. Acupuncture is a complex process, stimulation parameters, psychological effects of subjects, sociocultural, and contextual-environmental factors are factors that influence the effect of acupuncture. Therefore, better design is still needed to verify the accurate and specific connection.

In a word, the trend of MRI research on acupuncture mainly focuses on the functional connection of the brain, especially the limbic system and default network. However, in order to ensure the repeatability and reliability of the research results, the research design and quality control still need to be further strengthened.

### Limitations

There are still some limitations in this study that need to be addressed. First, due to the limitations of the Citespace software, we only analyze English articles from web of science database, which might lead to language and publication bias; Second, Citespace V is just a software to visualize and analyze networks, MRI analysis methods, the types of acupuncture, and the diseases involved need to be further analyzed in the future.

## Conclusion

In conclusion, this study provided an insight into MRI research on acupuncture with valuable information over 26 years. The current research status revealed that MRI study of acupuncture still has great development potential, and China needs to strengthen cooperation with other countries. The hot topics and trends of MRI research on acupuncture are mainly involved in the modulation of acupuncture on the limbic system, especially LPNN and DMN. Pain is the most frequently studied disease. However, in order to ensure the repeatability and reliability of the research results, the research design and quality control still need to be further strengthened.

## Data Availability Statement

The original contributions presented in the study are included in the article/supplementary materials, further inquiries can be directed to the corresponding author/s.

## Author Contributions

JZ and JX conceived the idea. YZ and LH collected the literature. XH, YL and JL conducted the data analysis. JZ drafted the manuscript. HY, QH, and JX revised the article. All authors have read and approved the final article.

## Conflict of Interest

The authors declare that the research was conducted in the absence of any commercial or financial relationships that could be construed as a potential conflict of interest.
